# The Anomalies of Hyaluronan Structures in Presence of Surface Active Phospholipids—Molecular Mass Dependence

**DOI:** 10.3390/polym10030273

**Published:** 2018-03-06

**Authors:** Piotr Bełdowski, Tomasz Andrysiak, Aleksandra Mreła, Zenon Pawlak, Wayne K. Augé, Adam Gadomski

**Affiliations:** 1 Institute of Mathematics and Physics, UTP University of Science and Technology, 85-796 Bydgoszcz, Poland; piotr.beldowski@utp.edu.pl (P.B.); adam.gadomski@utp.edu.pl (A.G.); 2 Faculty of Telecommunications, Computer Science and Technology, UTP University of Science and Technology, 85-796 Bydgoszcz, Poland; 3 Faculty of Technology, Kujawy and Pomorze University in Bydgoszcz, Toruńska 55-57, 85-023 Bydgoszcz, Poland; a.mrela@kpsw.edu.pl; 4 University of Economy, Biotribology Laboratory, Garbary 2, 85-229 Bydgoszcz, Poland; zpawlak@xmission.com; 5 Tribochemistry Consulting, Salt Lake City, UT 84117, USA; 6 Department of Research and Development, NuOrtho Surgical, Inc., Boston, MA 02723, USA; nnmoc@aol.com

**Keywords:** hyaluronan (A-), phospholipid, articular cartilage

## Abstract

Interactions between hyaluronan (A-) and phospholipids play a key role in many systems in the human body. One example is the articular cartilage system, where the synergistic effect of such interactions supports nanoscale lubrication. A molecular dynamics simulation has been performed to understand the process of formation of hydrogen bonds inside the hyaluronan network, both in the presence and absence of phospholipids. Additionally, the effect of the molecular mass of (A-) was analyzed. The main finding of this work is a robust demonstration of the optimal parameters (H-bond energy, molecular mass) influencing the facilitated lubrication mechanism of the articular cartilage system. Simulation results show that the presence of phospholipids has the greatest influence on hyaluronan at low molecular mass. We also show the specific sites of H-bonding between chains. Simulation results can help to understand how hyaluronan and phospholipids interact at several levels of articular cartilage system functioning.

## 1. Introduction

Surface active phospholipids play a vital role in biologic tissue systems, in large part due to an amphiphilic nature that allows for varied structural properties. In synovial joint organ systems, a surface active phospholipid layer (SAPL) covers normal articular surfaces in an oligolamellar structural formation [1,2,3,4,5]. The SAPL serves to integrate interfacial functions between juxtaposed surfaces and has been a subject of much inquiry due to its tribological features [6,7,8,9,10,11,12,13]. However, at sites of articular cartilage damage, the SAPL is deactivated, because a suitable substrate upon which a SAPL can form does not exist [1,6,7,14]. Hyaluronan (A-) is another key component in system lubrication, both as a viscosity modifier and for protecting SAPL [15,16,17]. The synergistic effect of both molecules’ interactions can provide (together with lubricin) conditions facilitating the lubrication of articular cartilage system [18,19,20].

**Figure 1:**
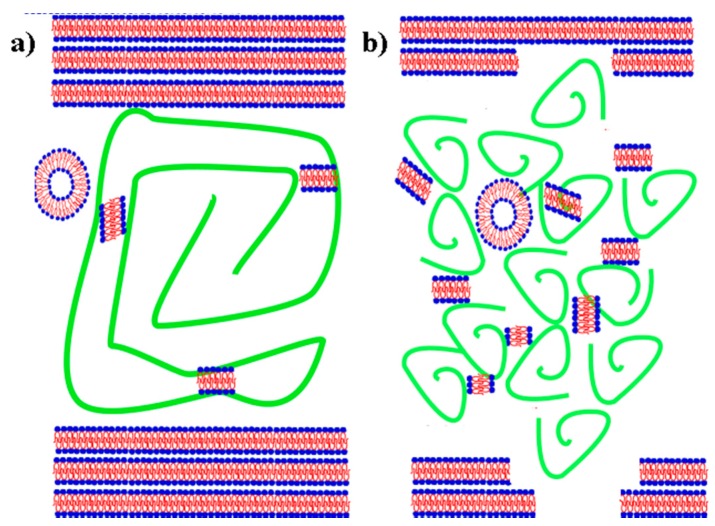
Artistic depiction of a model synovial joint organ system. The articular cartilage surface is represented as multilamellar bilayers of the SAPL without the underlying cartilage zones. Between the articulating surfaces, liposomal and lamellar phases are shown interacting with hyaluronan (A-) presented as molecular chains (green) in various states of cross-linking and network formation. Normal system conditions (of gel type) with an intact SAPL and long chain hyaluronan molecules (**a**) and pathological system conditions (of sol type) with a disrupted SAPL and broken hyaluronan molecules (**b**) are depicted.

During normal functioning, the SAPL serves as a sacrificial perturbation layer, whereby it can reform by self-assembly mechanisms after dissipation of incipient perturbation loads. In pathological synovial fluid, the hyaluronan structure changes [1,21,22] toward shorter chains. A simplified picture of the changes occurring during osteoarthritis (OA) changes is presented in Figure 1. As presented in the picture, synovial fluid components form two distinct structures regarding (A-):PL concentration. In a normal state, two components create separate structures interacting with each other to a greater or lesser extent depending on lubrication regime. Thus, the properties of both fluids are remarkably different—Newtonian vs. non-Newtonian (rheopectic) fluid for abnormal and normal synovial fluid (SF) respectively.

**Figure 2:**
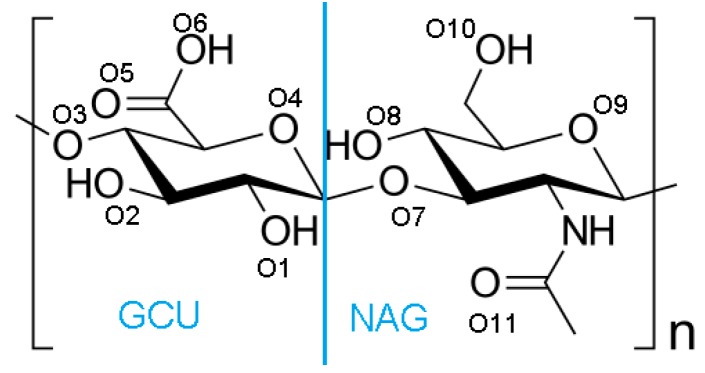
Structure of hyaluronan as composed of two parts GCU (d-glucuronic acid) and NAG (*N*-acetyl-d-glucosamine). Oxygen atoms are numbered to show H-bond formation between specific atoms.

As has been presented in several studies, to some extent, the flexibility of hyaluronan chains in solution is determined by the restricted rotation about the various glycosidic linkages [23]. On the other hand, the conformational freedom of the molecule is dependent on other factors, such as the presence of other molecular species such as DPPC. Hydrogen bonds between the glucuronyl carboxyl group and the adjacent acetamido hydrogen of the *N*-acetyl hexosamine ring contribute to chain stiffness [23] (see Figure 2), yet the number of studies on bonding inside network is sensitive on molecular mass [3,24]. Intra- and interchain interactions of the type described for (A-) are not uncommon in solutions of polysaccharides, and may be responsible for gel formation [23]. Complexes of (A-):PL show remarkable lubricating properties [25]; however, the nature of their interactions has not been fully understood, and therefore, the present study focuses on one of the most important factors in gel formation.

The purpose of this study was to evaluate molecular interactions by hydrogen bonding between hyaluronan (A-) and dipalmitoylphosphatidylcholine (DPPC) molecules. Molecular dynamic simulations of the hydrogen bond formation (–OH···PO_4_^−^) moieties between hyaluronan with molecular weights of 10, 40, and 160 kDa and phospholipid were performed.

## 2. Materials and Methods

All atom molecular dynamic simulations were performed using AMBER03 force field in YASARA Structure Software (YASARA Biosciences GmbH, Vienna, Austria) to evaluate interactions between hyaluronan macro-ion and phospholipid molecules. A- and PL structures were downloaded from PubChem (Open Chemistry DataBase, Bethesda, MD, USA). The lipid used for this study was dipalmitoylphosphatidylcholine (DPPC). The hyaluronan macro-ion was modified to obtain longer chains by using YASARA Structure Software. The final molecular masses of (A-) were: 10, 40 and 160 kDa; additionally, a TIP4P water model was used. The isobaric-isothermal ensemble, all atom simulations were performed under the same conditions: temperature 310 K, at pH = 7.0 and at 0.9% NaCl aqueous solution, with a time step of 2 fs. Berendsen barostat and thermostat with a relaxation time of 1 fs were used to maintain constant temperature and pressure. Then the DPPC solution was mixed and added at a concentration of *_C_*_PL_ = 20·10^−8^ *M*, i.e., much higher than the *cmc* of DPPC *c*_cmc_~ 5 × 10^−10^ *M*. The final concentration of (A-) was *c*_A_*^−^* = 10^−6^ *M*—much higher than in SF; however, we focus more on the dense network region of SF, rather than the overall concentration. For molecular mass dependence on (-A···PL) interactions, two types of samples were tested: (i) (A-) only, and (ii) (A-) with phospholipid.

The concentrations of dipalmitoylphosphatidylcholine were taken to show the effect of (similarly to works [26,27]). To describe a process of cross-linking, we used several variables described below. 

Radius of gyration *R*_g_ is defined by Equation (1) as the root mean square distance of the atoms from the center of mass as 

(1) $$R_{g}=\sqrt{\frac{1}{N}\sum_{i=1}^{N}{\left(C-R_{i}\right)}^{2}.}$$

We consider total and intermolecular bonds separately: total is a sum of interactions between all atoms of (A-), whereas intermolecular is a sum of interactions between separate chains; thus, it does not exist for long (A-), because all interactions take place within a single chain. Thus, hydrogen bond energy is defined as $E_{\mathrm{total}}=E_{\mathrm{intermoleclar}}+E_{\mathrm{intramolecular}}$ Solvent-accessible surface, as evaluated in this study, consists of all the points that the center of the water probe (i.e., the nucleus of the oxygen atom in the water molecule) can reach while rolling over the solute; the procedure of calculating this variable has been presented in [28,29]. The molecular structure of hyaluronan is presented in Figure 2. 

### Hydrogen Bond Identification and Strength

Hydrogen is formed between two oxygen atoms if: (i) the distance between the hydrogen and adjacent oxygen atoms is smaller than $R_{O-H}$; and (ii) the distance between two neighboring oxygen atoms is less than $R_{O-O}$. Hydrogen bond energy, as defined by Equation (2), is greater than 6.25 kJ/mol (or 1.5 kcal/mol), which is 25% of the optimum value 25 kJ/mol. Thus, only strong and weak (up to 2.6 Å) are considered in the analysis. Equation (2) yields the bond energy in kJ/mol as a function of the Hydrogen-Acceptor distance and two scaling factors:

(2) $$E_{\mathrm{HB}}=25\cdot \frac{2.6-\max\left(Dis_{H-A,}2.1\right)}{0.5}\cdot Scale_{D-A-H}\cdot Scale_{H-A-X}$$

where the first scaling factor depends on the angle formed by Donor-Hydrogen-Acceptor, and the second scaling factor is derived from the angle formed by Hydrogen-Acceptor-X, where the latter X is the atom covalently bound to the acceptor. A more detailed definition of the scaling factors $Scale_{D-A-H}$ and $Scale_{H-A-X}$ has been presented in [26]. If the Acceptor forms more than one covalent bond, the one with the lowest scaling factor is taken. YASARA assigns at most one hydrogen bond per hydrogen atom, picking the better one if two acceptors are available. 

## 3. Results

The presented results are divided into three parts, describing separate effects of (-A···PL) interactions. Namely, (i) (A-) cross-linking dependence on its molecular mass; (ii) cross-linking of (A-) dependence of its concentration; (iii) influence of those two effects on (-A···PL) hydrogen bond formation of hyaluronan-hyaluronan molecular (-A···A-) interaction. 

### 3.1. Influence of Hyaluronan Molecular Mass on Cross-Linking in Presence of PL

**Figure 3:**
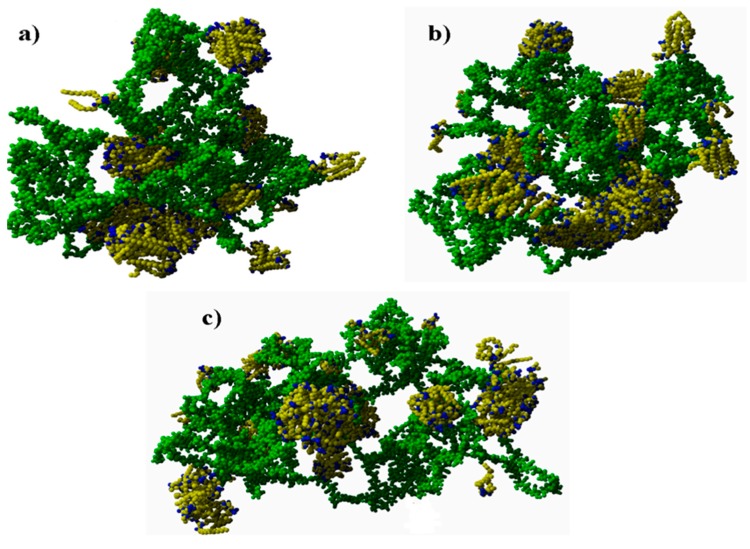
Final structures obtained from the simulation for different (A-) molecular masses (MM). From left to right the (A-) molecular mass increases. Hydrophilic parts of PL are depicted as blue, whereas their hydrophobic counterparts—yellow. (A-) is depicted as green for better visualization of PL orientation towards the (A-) network. Structures show three cases for MM of hyaluronan: (**a**) short 10 kDa; (**b**) mid 40 kDa; and (**c**) long 160 kDa.

Final structures for different molecular masses (MM) are presented in Figure 3. The simulations showed that the longer the chain was, the smaller the PL structures created. For the longest chain, many smaller micelles were present, whereas medium and shorter chains were surrounded and/or penetrated by quasi-bilayers. Phospholipid was oriented toward (A-) in two ways: (i) with their hydrophilic part, connected to H-bond formation between oxygen atoms; and (ii) with their hydrophobic parts connected to hydrocarbon-hydrocarbon bonding.

**Figure 4:**
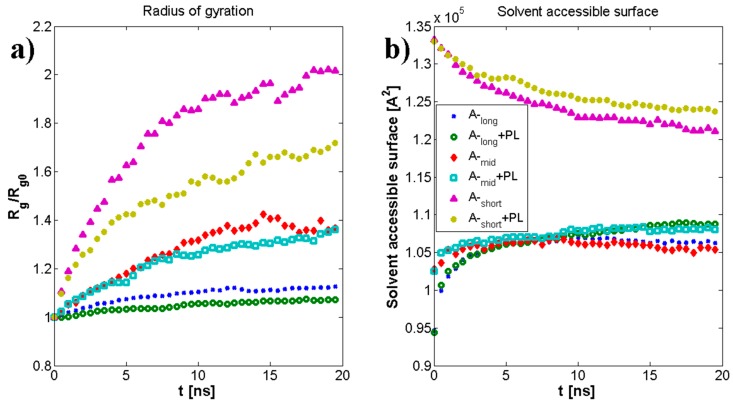
(**a**) The normalized radius of gyration as divided by its initial value and (**b**) solvent-accessible surface of (A-) chain for three molecular masses (MM) with and without phospholipid.

**Figure 5:**
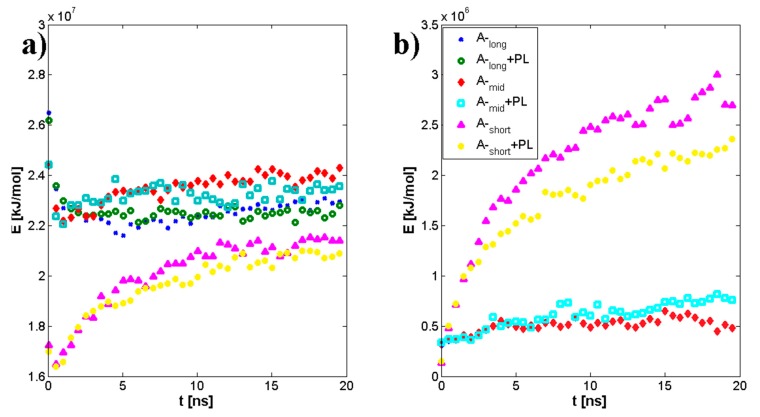
Total hydrogen bonds energy inside hyaluronan (A-) network for three molecular masses: (**a**) total hydrogen bonds energy; (**b**) intermolecular hydrogen bonds energy.

**Figure 6:**
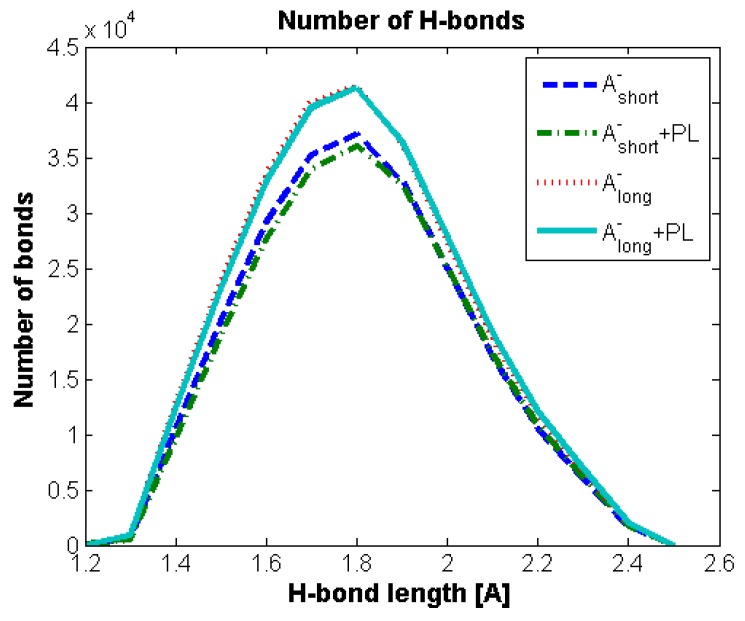
Total number of H-bonds between A- chains dependent on H-bond length.

**Figure 7:**
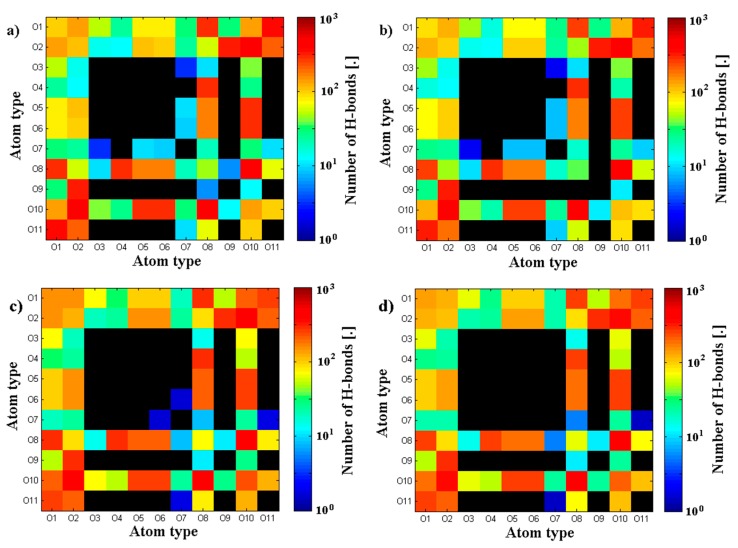
Map of average H-bond length between specific oxygen atoms of (A-) chains. Two cases of short (A-) are presented: (**a**) short (A-) solution; (**b**) short (A-) solution + PL; (**c**) long (A-) solution; (**d**) long (A-) solution + PL. Black color indicates no H-bond found between two atoms.

**Figure 8:**
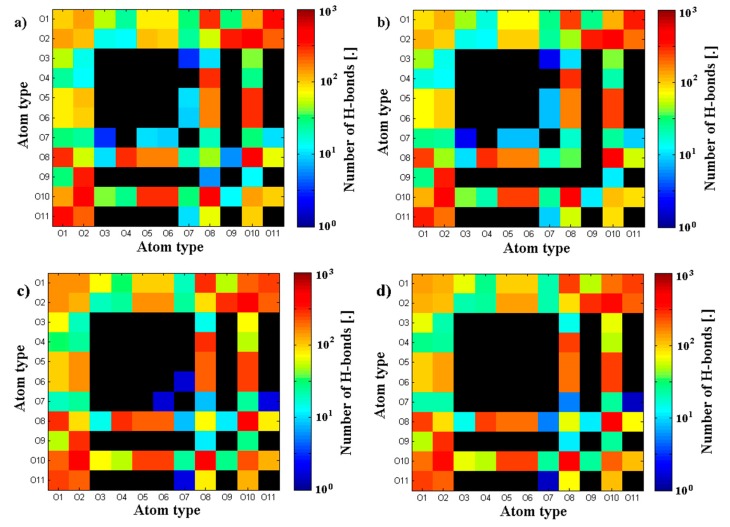
Map of H-bond number $N_{\mathrm{Hbond}}$ between specific oxygen atoms of (A-) chains. Two cases of short (A-) are presented: (**a**) short (A-) solution; (**b**) short (A-) solution + PL; (**c**) long (A-) solution; (**d**) long (A-) solution + PL. Black color indicates no H-bond found between two atoms.

Geometrical properties are presented in Figure 4. Namely, Figure 4a depicts the evolution of radius of gyration as a function of time. The results are in good agreement with previously presented numerical and experimental data [24]. The highest impact on separate molecular mass has been seen for the shortest chains. The longer the chain is, the smaller the influence on its size. As described by [24] for (A-) chains longer than 170 kDa, both molecules change their structure from micelles absorbing (A-) chains to cylinders. Figure 4b shows the penetration of the (A-) structure by water molecules. There is an apparent distinction between short and medium/long chains, as the total surface is between 20% and 30% higher, which could also be related to self-affiliation of the chains and higher degrees of freedom. Figure 5a,b presents the total and intermolecular hydrogen bond energy, respectively, inside the (A-) network. There is a link between the radius of gyration evolution and H-bond energy. Namely, expanding chains form more H-bonds. This is mostly visible for short chains, in the case of longer chains, (A-) is more stable and there is more exchange of H-bonds inside the network, rather than formation of new ones. Short chains are able to diffuse in the system more freely; thus, the intermolecular H-bond energy is more significant in this case and the addition of PL causes the competition of H-bond formation between (-A···A-) and (-A···PL) bonds. Longer chains are less penetrable by lipids due to the higher number of (-A…A-) interactions inside the chains. Figure 6, Figure 7 and Figure 8 show the molecular detail of hydrogen bonds (-A···A-) for two types of chains: long and short. As presented in Figure 6, distribution of H-bond strength is similar in all cases. However, the longer chains show higher overall numbers of H-bonds. Both very strong (*r*_H__₋bond_ < 1.5) and weak (*r*_H__₋bond_ > 2.2) H-bonds represent ~10% of all bonds, strong H-bonds (1.5 < *r*_H__₋bond_ < 2.2) represent 80% of all bonds. The addition of PL changes characteristics of specific H-bonds as presented in Figure 7 and Figure 8. Moreover, long and short chains have slightly different H-bond distributions, after adding PL, weak and infrequent bonds disappear.

### 3.2. Hyaluronan-Phospholipd Bonding

**Figure 9:**
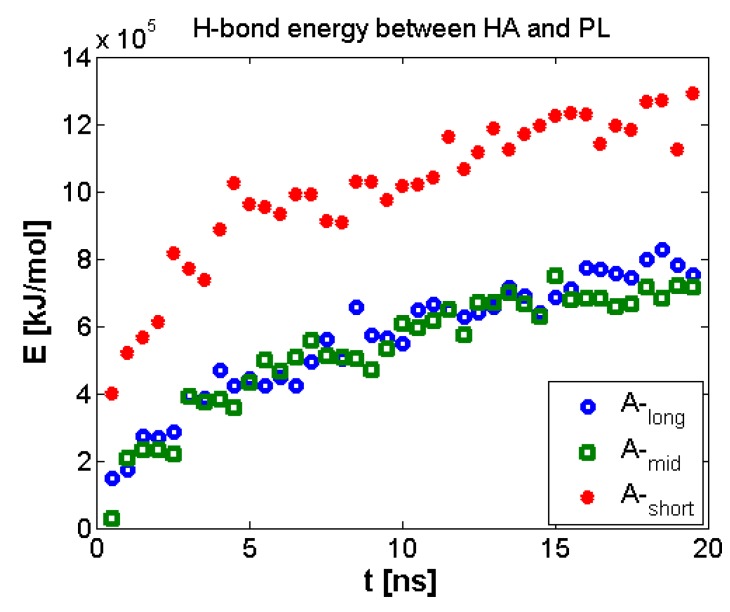
Normalized H-bonds energy between (A-) chains for all lengths with phospholipid.

Finally, we look at the bond creation between A- and PL, namely, hydrogen bonds formation between (–OH···PO_4_^−^), as presented in Figure 9.

## 4. Discussion

Pathological changes occurring in OA synovial fluid [22] are connected to a three-fold increase in PL concentration. Additionally, there is a change of polydispersity of (A-), due to the higher rate of polymer degradation. Taking this into account, we are trying to understand the mechanism of maintenance of physical properties of (A-) network under pathological condition. Previously presented studies [24,30,31] have shown that short (A-) decreases its geometrical size with the higher PL concentration. The hyaluronan polymer chain, which may contribute to the unusual rheological properties, makes them ideal as a supporting lubricant. In mammals, the enzymatic degradation of (A-) results from the action of the enzymes. As presented in [24,32,33,34], the interaction is mainly governed by (A-) molecular mass. Namely, (A-) chains shorter than 170 kDa are adsorbed onto PL vesicles, whereas longer chains create cylindrical forms. The other goal was to understand the nature of the transition phase of the formation of (-A···PL) complexes.

Our data present a possible mechanism for (-A···PL) interaction in normal and pathological conditions. Figure 4a presents the evolution of the radius of gyration of an average (A-) chain. As one can see, PL mostly affects short chains of A-, the difference between the pure solution and one in the presence of PL is ~20%, whereas for medium and long chains it is 10% and 5%, respectively. The solvent-accessible surface presented in Figure 4b shows that short A- has on average the higher penetration of water, which can be explained by the higher surface area of fragmented hyaluronan polymer chain.

Figure 5, Figure 6, Figure 7 and Figure 8 present the cross-linking abilities of (A-), namely, the H-bond formation of intra and intermolecular networks. Shorter chains are able to form intermolecular networks, as they show much higher diffusivity than longer chains. On the other hand, total H-bond energy is higher for long chains, which creates denser intramolecular networks. These denser networks are able to repel PL from the interior and function as a better supporting macromolecule lubricant. Figure 7 and Figure 8 show that there are some differences between long and short chains in term of the specific H-bonding site. Overall, both structures have a very similar distribution of bonding sites; however, the addition of phospholipids tends to decrease the number of least frequent bonds to form. 

The bonding of (A-) with PL presented in Figure 9 shows that shorter chains create more contacts with PL. This would indicate the competition between (-A···A-) and (-A···PL) contact formation. (A-) plays its supporting role best when the network is well established; thus, high penetration of PL is a weakening factor in the described phenomenon.

The changes in the setting of synovial fluid are predominantly outcomes of the latter process (e.g., occurrence of changeable structures of hyaluronan). The changes influence the existence of friction regimes in joint systems [35]. For further study, we assume which changes are most often of anomalous types, i.e., they were created as a result of some alter type mechanism working and, considering their three-dimensional character, they significantly differ from their surroundings or from the whole structure. The hyaluronan-polymer in pH 7.3 SF assumes a stiff helical configuration, which can be attributed to hydrogen bonding between the hydroxyl groups of hyaluronan (A-) and (–PO_4_^−^) of PLs along the chain. Rheopexy of SF, an anomalous viscoelastic phenomenon of stress increase upon time raising at shear rate stationarity, has also been addressed in terms of protein aggregations by [36].

## 5. Conclusions

In this study, we have shown the interaction between (–OH···PO_4_^−^) as dependent on (A-) molecular mass. Negatively charged DPPC were tested for their interaction with long, medium and short chains of hyaluronan (A-) M.W. 160, 40 and 10 kDa at pH ~ 7. The formation of phospholipid membrane structures has been shown to be supported by (A-) long molecules; in the presence of low molecular (or short chain) (A-), formation of liposomes and lamellar phases may be less efficient. The decreased friction coefficient demonstrated by some investigators indicates that DPPC could complement the boundary-lubricating ability of long molecules of hyaluronan in joints, which is demonstrated in this work. We also explained how hyaluronan (A-) could have a carrier function for insoluble phospholipid, in the molecular bonding of (A-) and DPPC; namely, hydrogen bond formation between (–OH···PO_4_^−^) groups.
